# Cortical metabolic characteristics of anti-leucine-rich glioma-inactivated 1 antibody encephalitis based on ^18^F-FDG PET

**DOI:** 10.3389/fneur.2023.1100760

**Published:** 2023-03-31

**Authors:** Kai Wang, Xiaobin Zhao, Leilei Yuan, Qian Chen, Qun Wang, Lin Ai

**Affiliations:** ^1^Department of Nuclear Medicine, Beijing Tiantan Hospital, Capital Medical University, Beijing, China; ^2^Department of Neurology, Beijing Tiantan Hospital, Capital Medical University, Beijing, China

**Keywords:** LGI1, positron emission tomography, encephalitis, fluorodeoxyglucose, cortical metabolism

## Abstract

**Purpose:**

A general glucose metabolism pattern is observed in patients with anti-leucine-rich glioma-inactivated 1 (LGI1) antibody encephalitis; however, it is unclear whether further subregional metabolic differences exist. Therefore, the present study aimed to conduct an in-depth exploration of the features of glucose metabolism within specific brain areas using ^18^F-fluorodeoxyglucose positron emission tomography (^18^F-FDG PET).

**Materials and methods:**

This retrospective study enrolled thirteen patients confirmed with LGI1 antibody encephalitis who were admitted to Beijing Tiantan Hospital from June 2021 to September 2022. All patients underwent ^18^F-FDG PET before initiating clinical treatment. Changes in glucose metabolism in specific brain areas were analyzed using Cortex ID software. The laterality of ^18^F-FDG uptake was assessed, and differences in specific brain areas were compared using paired *t*-tests.

**Results:**

Significant metabolic changes in at least one brain region in 11 out of 13 patients (84.6%) were revealed by semi-quantitative analysis (*z*-score > 2). A bilateral decrease in the ^18^F-FDG metabolic pattern was revealed in almost all brain regions of interest; in contrast, a hypermetabolic pattern was observed in the medial temporal region, with mean *z*-scores of 1.75 ± 3.27 and 2.36 ± 5.90 on the left and right sides, respectively (*p* = 0.497). In the prefrontal and temporal lobes, ^18^F-FDG metabolism was significantly lower in the lateral region than in the medial region on both sides. For the cingulate cortex, significant hypometabolism was also observed in the posterior part compared to the anterior counterpart on both the left (*z*-score: −1.20 ± 1.93 vs. −0.42 ± 1.18, respectively; *p* = 0.047) and right (*z*-score: −1.56 ± 1.96 vs. −0.33 ± 1.63, respectively; *p* = 0.001) sides. However, a significant difference in regional metabolism was observed only on the left side (*p* = 0.041).

**Conclusion:**

An asymmetric ^18^F-FDG metabolic pattern exists in patients with anti-LGI1 encephalitis. Meanwhile, varied regional metabolic differences were revealed bilaterally in specific cerebral areas, which could be associated with the clinical manifestations.

## 1. Introduction

Leucine-rich glioma-inactivated 1 (LGI1) antibody encephalitis is a type of autoimmune encephalitis that affects the central nervous system. It is characterized by inflammation of the brain, especially the limbic system and basal ganglia, which is responsible for in progressive memory alteration, psychiatric symptoms, and faciobrachial dystonic seizures (FBDS) (1, 2). The exact mechanism of anti-LGI1 encephalitis is not fully understood, but it is thought to involve the production of autoantibodies against LGI1 proteins that is located on the neuronal surface, the condition is more common presented in men and typically affects individuals over the age of 50, although it can occur at any age (3–5). Normally, LGI1 proteins are important for regulating the communication between neurons in the brain. In LGI1 encephalitis, antibodies bind to the LGI1 proteins on the surface of neurons, leading to inflammation and damage to brain tissue. The inflammation caused by the autoantibodies leads to a variety of symptoms such as seizures, memory loss, confusion, and changes in behavior. The antibodies can also interfere with the normal function of LGI1 proteins, which can further disrupt communication between neurons in the brain.

In clinical practice, the clinical diagnosis of anti-LGI1 encephalitis mainly relies on clinical symptoms, antibody testing, and magnetic resonance imaging (MRI). In addition to the T2-weighted imaging/fluid-attenuated inversion recovery (T2WI/FLAIR) hyperintensities on MRI scans, ^18^F-fluorodeoxyglucose positron emission tomography (^18^F-FDG PET) is a potential marker for anti-LGI1 encephalitis (6, 7), In some cases, MRI may not detect abnormalities in patients with anti-LGI1 encephalitis, particularly in the early stages of the disease. In contrast, ^18^F-FDG PET can detect changes in glucose metabolism in the brain that may not be apparent on MRI. ^18^F-FDG PET is able to measure brain metabolism and perform quantitative assessment which may be particularly helpful in monitoring disease progression and response to treatment over time. Unlike antibody testing, in addition, ^18^F-FDG PET is a non-invasive imaging technique that does not require invasive procedure that could be more sensitive to pathological alterations than assessments based on electroencephalography and inflammatory marker detection in the cerebrospinal fluid (8). However, the manifestation of changes in ^18^F-FDG metabolism are dependent on various factors, such as the patient’s clinical history and the status of corticosteroid treatment. Asymmetric uptake within the hippocampus and basal ganglia was previously found in patients with anti-LGI1 encephalitis (9–11). In addition, asymmetric LGI1 expression has been observed between bilateral globus pallidus and hippocampal samples (11). These findings suggest that the clinical symptoms and functional impairment that occur may be associated with varied metabolic patterns. Further exploration of the pattern of ^18^F-FDG metabolism in specific brain regions or functional cortices in anti-LGI1 encephalitis could provide novel insight for understanding and interpreting the clinical manifestations of the disease, and could also provide novel clues for monitoring therapeutic effects in patients.

Previous studies on anti-LGI1 encephalitis using ^18^F-FDG PET have mainly focused on the metabolic alterations occurring within entire cerebral lobes (temporal, parietal, or frontal lobe), deep nuclei, or the hippocampus (12–15). The present study aimed to assess the laterality of changes in cortical metabolism and the patterns within specific sub-lobar regions among patients with anti-LGI1 encephalitis *via* analysis using Cortex ID software to promote our understanding of the varied clinical manifestations associated with regional metabolic abnormalities.

## 2. Materials and methods

### 2.1. Patients

A total of 13 patients diagnosed with LGI1 antibody encephalitis were recruited in this retrospective cohort study between June 2021 and September 2022 at Beijing Tiantan Hospital. The present study was approved by the Ethics Committee of Beijing Tiantan Hospital, Capital Medical University. The requirement for written informed consent was waived due to the retrospective nature of the study. Patients were enrolled according to the following inclusion criteria: (1) anti-LGI1 encephalitis was first diagnosed at our institute; (2) detailed and complete records describing clinical characteristics were available; (3) a relatively acute or subacute disease course was presented; and (4) ^18^F-FDG PET had been performed before the initiation of clinical treatment. Two neurological experts (Z.X.B. and Y.L.L.) diagnosed anti-LGI1 encephalitis according to published consensus criteria (6).

### 2.2. LGI1 antibody detection

Serum and cerebrospinal fluid samples were collected from all patients for antibody detection, including LGI1, N-methyl D-aspartate receptor, contactin-associated protein 2, α-amino-3-hydroxy-5-methyl-4-isoxazolepropionic acid receptor, and γ-aminobutyric acid type B. Both sample types were tested for the presence of LGI1 antibodies as described in our previous study (15). The presence of antibody in serum and cerebrospinal fluid samples was determined based on the judgment of two experienced investigators.

### 2.3. Brain ^18^F-FDG PET protocol

All participants underwent a brain imaging evaluation before clinical treatment was initiated. ^18^F-FDG PET examination was performed in 12 of the 13 patients using a PET/computed tomography (CT) scanner (Discovery Elite, GE Healthcare) from which the brain images were obtained, whereas the remaining patient underwent examination *via* PET/MRI (Discovery 750w, GE Healthcare). Sedative drugs were not used in any of the patients before the PET image acquisition. Patients received intravenous injection of ^18^F-FDG at a dose of 3.7–5.0 MBq/kg after fasting for at least 6 h. The blood glucose levels were confirmed to be no higher than 8 mmol/L before injection. Participants kept a quiet resting status without light stimulation during the uptake period. PET acquisitions started after 45–60 min post-injection for brain examinations. 3D-time-of-flight mode was applied during the PET image acquisition over a 10-min period. For PET/CT, the brain imaging data were further processed using ordered subset expectation maximization methods, with four iterations and eight subsets, with smoothing using a 5-mm full-width at half-maximum filter. For PET/MRI, T1-weighted images, T2WI, FLAIR, and contrast-enhanced T1WI images were acquired based on the standard acquisition procedure. Attenuation correction of the ^18^F-FDG PET images was performed using the zero echo time method.

The ^18^F-FDG PET images were reconstructed by using the GE Advanced Workstation 4.6 software package (GE Healthcare). Two senior nuclear medicine physicians (C.Q. and A.L.) with more than 10 years of diagnosis experience visually assessed and interpreted the PET results. Abnormalities of glucose metabolism in the cortical regions, hippocampus, and basal ganglia were carefully assessed. Brain MRI results were evaluated by two experienced neuroradiologists (C.Q. and W.K.), who were blinded to each patient’s clinical information. In this study, T1WI, T2WI, FLAIR, and contrast-enhanced T1WI signals were also reviewed by experts who were blinded to the patients’ clinical diagnoses. Data from each patient was categorized as either normal or abnormal by consensus; any inconsistent diagnosis was further discussed to achieve a confirmed result.

### 2.4. Processing of PET data

After visual assessment, ^18^F-FDG PET images were imported into the Cortex ID suite (GE Healthcare) on a workstation for metabolic analysis of the cortex. ^18^F-FDG uptake in cortical regions was demonstrated through 3D stereotactic surface projections, which can provide a surface-rendered display in addition to the conventional perspective of axial, sagittal, and coronal images. Cortical glucose metabolic alterations were normalized to the pons, and a metabolic map of the whole brain surface was created. Automated voxel-by-voxel *z*-scores generated by the Cortex ID software were calculated bilaterally for the following regions of interest: prefrontal cortex (lateral and medial), sensorimotor cortex, cingulate cortex (anterior and posterior), precuneus cortex, parietal cortex (superior and inferior), lateral occipital cortex, primary visual cortex, and temporal cortex (lateral and medial). The following formula was used for *z*-score calculations:


$$z-\mathrm{score}=\left[\mathrm{mean subject}-\mathrm{mean database}\right]/\mathrm{SD}\;\mathrm{database}$$

For each specific brain region, voxel-based color coding was used to depict the mean *z*-scores of the range of the metabolic alterations. In this semiquantitative analysis, a *z*-score threshold higher than 2 (≃1.96 based on a two-tailed test), which corresponds to a *p* value of 0.05 (two-tailed), was regarded as the cutoff for significant abnormalities, with positive *z*-scores being indicative of hypermetabolism compared to the pons. All values were validated by visual inspection.

### 2.5. Statistical analysis

Paired *t*-tests were used to compare metabolic differences between bilateral or regional brain areas using SPSS Statistics software 23.0 software package for Windows (IBM Corp., Armonk, NY). Statistical significance was set at *p* < 0.05 (two-tailed).

## 3. Results

### 3.1. Characteristics of the patient population

Thirteen patients with clinically confirmed anti-LGI1 encephalitis were included (seven men and six women; median age, 57 years). The clinical information of the patients are summarized in Table 1. All patients manifested with either impaired neurological function, including seizures (10/13, 76.9%), abnormal limb activity (4/13, 30.8%), impaired memory (8/13, 61.5%), and speech disorder (3/13, 23.1%). All 13 (100%) patients tested positive for LGI1 antibodies, in both the serum and cerebrospinal fluid (76.9%), with three (23.1%) patients showing only in the cerebrospinal fluid or serum. The patients tested negative for other antibodies of interest.

**Table 1:** Clinical characteristics of the patients included in the study.

Patient no.	Age (years)	Gender	Impaired neurological function				Duration from symptom onset to ^18^F-FDG PET (weeks)	Type of ^18^F-FDG PET	Hypermetabolism of bilateral basal ganglia and/or thalamus
			Seizures	Abnormal limb activity	Impaired memory	Speech disorder			
1	77	F	+	−	−	−	3	PET/CT	Yes
2	53	M	+	+	+	+	24	PET/CT	Yes
3	30	F	−	–	+	−	2	PET/CT	Yes
4	67	M	+	+	−	−	12	PET/CT	Yes
5	77	F	−	−	+	−	2	PET/CT	Yes
6	55	M	−	−	+	+	8	PET/CT	Yes
7	60	M	+	−	+	−	24	PET/CT	Yes
8	40	F	+	−	+	−	4	PET/CT	Yes
9	50	F	+	−	−	−	8	PET/MR	No
10	57	M	+	−	+	−	20	PET/CT	No
11	76	M	+	−	−	−	4	PET/CT	Yes
12	81	M	+	+	+	+	4	PET/CT	Yes
13	21	F	+	+	−	−	4	PET/CT	Yes

### 3.2. Imaging findings

A total of 13 brain ^18^F-FDG PET datasets were acquired from the enrolled patients with autoantibody levels appropriate for diagnosis. The median duration of symptoms before PET scanning was 4 weeks (interquartile range, 12.5 weeks). The results of the visual and semiquantitative assessments of cortical ^18^F-FDG uptake are shown in Supplementary Table 1.

**Figure 1:**
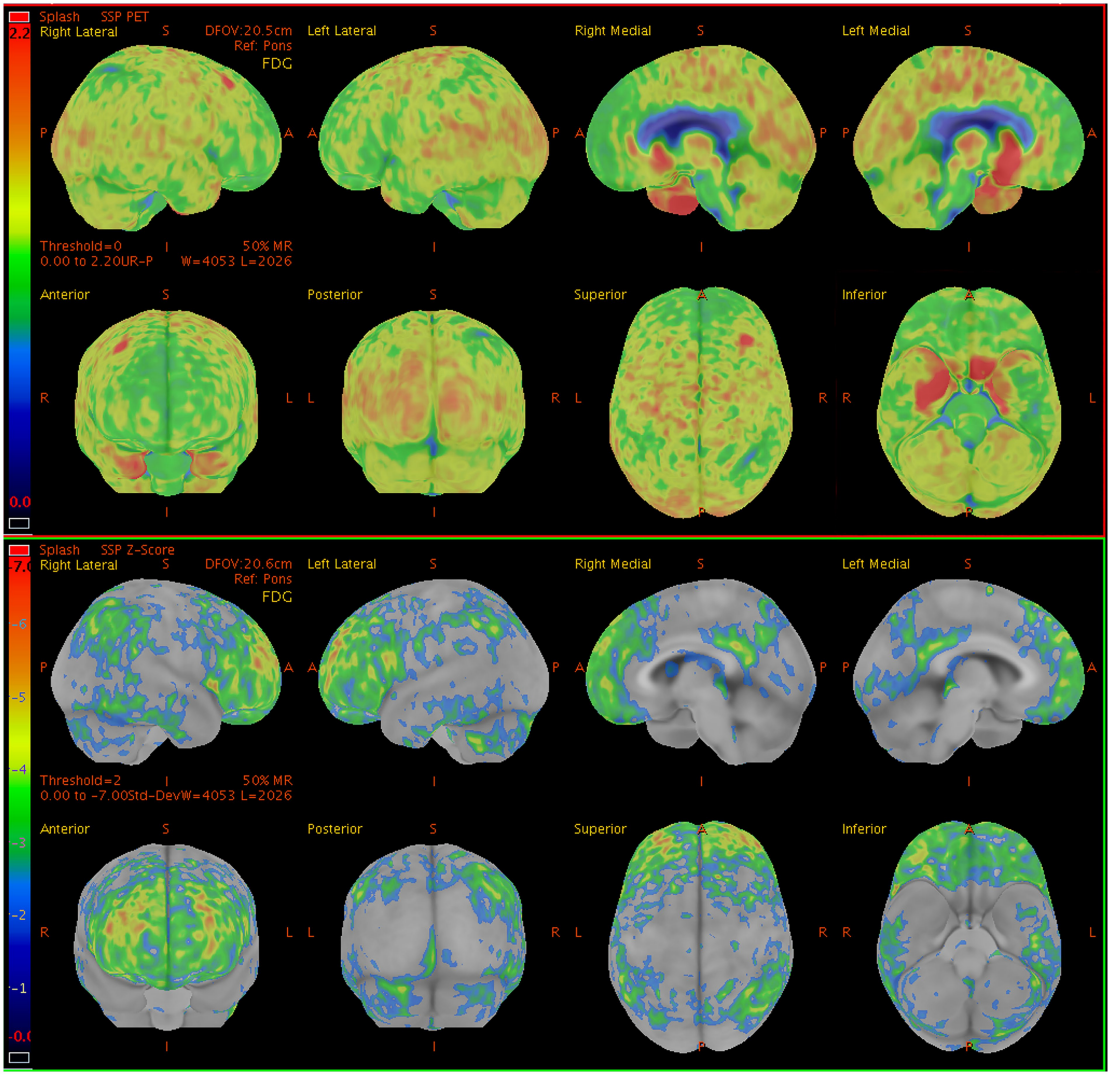
Sample brain ^18^F-FDG PET images from a 60-y-old man presenting with seizures, and memory impairment. The brain ^18^F-FDG PET *z*-score maps demonstrate significant hypometabolism in the lateral/medial prefrontal lobe (R, L), posterior cingulate gyrus (R, L), precuneus (R, L), superior parietal lobe (R), inferior parietal lobe (R, L), lateral occipital lobe (R, L), primary visual cortex (R, L), and lateral temporal lobe (R) relative to metabolic activity in the pons. In contrast, significant hypermetabolism is visible in the medial temporal lobe (R, L). Color bar represents the scale of the *z*-scores. ^18^F-FDG PET, ^18^F-fluorodeoxyglucose positron emission tomography; R, right side; L, left side.

Abnormal patterns of ^18^F-FDG uptake were identified in all 13 patients upon visual inspection, presenting as a varying decrease or increase in radiotracer uptake in the bilateral cortical hemispheres. Significant metabolic changes in at least one brain region in 11 out of 13 patients (84.6%) were revealed by the semi-quantitative analysis (*z*-score > 2). Lobar hypometabolism was the predominant abnormality on brain ^18^F-FDG PET images (Figure 1) and was observed in all patients. MRI findings were suggestive of LGI1 antibody encephalitis in patients who underwent ^18^F-FDG PET/MRI. Positive MRI findings revealed an hyperintensity on T2/FLAIR sequences in the medial temporal lobe, whereas there were no findings suggestive of LGI1 antibody encephalitis on the CT scans.

### 3.3. Bilateral differences in regional brain metabolism

**Figure 2:**
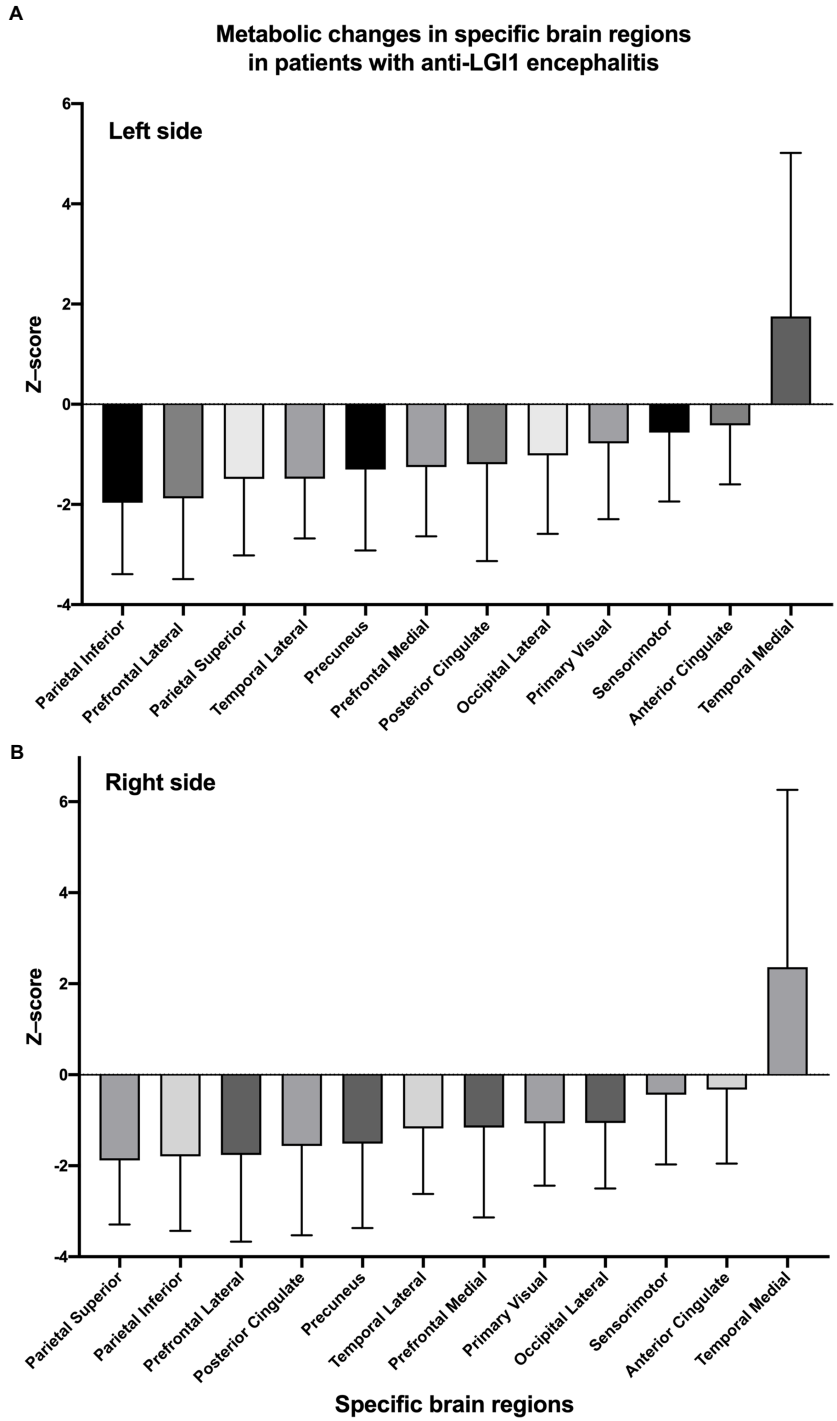
*Z*-scores of changes in glucose metabolism within specific brain regions on the left **(A)** and right **(B)** sides. Regions are sorted by mean *z*-scores in ascending order.

Overall, as determined using Cortex ID software, a decreased ^18^F-FDG metabolic pattern was evident bilaterally in almost all of the brain regions of interest. In contrast, the medial temporal region exhibited a hypermetabolic pattern, with mean *z*-scores of 1.75 ± 3.27 and 2.36 ± 5.90 on the left and right sides, respectively; however, they did not significantly differ (*p* = 0.497) (Figure 2). For the posterior cingulate region, the level of metabolic activity was significantly lower on the left side than on the right (−1.56 ± 1.96 vs. −1.20 ± 1.93, respectively; *p* = 0.045); however, no significant difference was observed for the other brain regions on both sides (Table 2).

**Table 2:** Bilateral comparison of regional ^18^F-FDG metabolism.

Brain regions	*Z*-score (mean ± SD)		*p*-value*
	Right	Left	
Prefrontal lateral (PFL)	−1.76 ± 1.91	−1.88 ± 1.61	0.539
Prefrontal medial (PFM)	−1.16 ± 1.98	−1.25 ± 1.38	0.715
Sensorimotor (SM)	−0.44 ± 1.53	−0.57 ± 1.37	0.485
Anterior cingulate (AC)	−0.33 ± 1.63	−0.42 ± 1.18	0.677
Posterior cingulate (PC)	−1.56 ± 1.96	−1.20 ± 1.93	0.045
Precuneus (P)	−1.51 ± 1.86	−1.30 ± 1.62	0.289
Parietal superior (PS)	−1.88 ± 1.41	−1.49 ± 1.53	0.148
Parietal inferior (PI)	−1.79 ± 1.64	−1.97 ± 1.43	0.319
Occipital lateral (OL)	−1.06 ± 1.44	−1.02 ± 1.57	0.856
Primary visual (PV)	−1.07 ± 1.37	−0.78 ± 1.52	0.237
Temporal lateral (TL)	−1.18 ± 1.44	−1.49 ± 1.19	0.068
Temporal medial (TM)	2.36 ± 3.90	1.75 ± 3.27	0.497

*Compared *via* paired *t*-test; *p* < 0.05 is considered statistically significant. ^18^F-FDG, ^18^F-fluorodeoxyglucose positron emission tomography; SD, standard deviation.

### 3.4. Positional differences in metabolism in certain brain regions

Because the analysis of bilateral differences failed to reveal certain significant findings, further analysis was conducted, focusing on differences in positional metabolism in specific brain regions. In the prefrontal and temporal lobes, ^18^F-FDG metabolism in the lateral region was significantly lower than that in the medial regions for both cerebral hemispheres (Table 3). In addition, significant hypometabolism was also observed in the posterior cingulate region compared to that of the anterior counterpart on the left side (*z*-score: −1.20 ± 1.93 vs. −0.42 ± 1.18, respectively; *p* = 0.047); a similar difference was observed on the right side (*z*-score: −1.56 ± 1.96 vs. −0.33 ± 1.63, respectively; *p* = 0.001). These findings indicated a much larger gap in metabolic activity between the posterior and anterior cingulate regions. As for the parietal lobe, significant hypometabolism was revealed in the inferior parietal region compared to that of the superior parietal region on the left side (*z*-score: −1.97 ± 1.43 vs. −1.49 ± 1.53, respectively; *p* = 0.041). Uptake of the radiotracer was slightly lower in the superior parietal region; however, the difference failed to show statistical significance (*p* = 0.785).

**Table 3:** Positional metabolic comparison of ^18^F-FDG metabolism in specific brain regions.

Brain regions	Side	Specific area		*p*-value*
		Lateral	Medial	
Prefrontal lobe	L	−1.88 ± 1.61	−1.25 ± 1.38	0.002
	R	−1.76 ± 1.91	−1.16 ± 1.98	0.003
		Anterior	Posterior	
Cingulate cortex	L	−0.42 ± 1.18	−1.20 ± 1.93	0.047
	R	−0.33 ± 1.63	−1.56 ± 1.96	0.001
		Superior	Inferior	
Parietal lobe	L	−1.49 ± 1.53	−1.97 ± 1.43	0.041
	R	−1.88 ± 1.41	−1.79 ± 1.64	0.785
		Lateral	Medial	
Temporal lobe	L	−1.49 ± 1.19	1.75 ± 3.27	0.005
	R	−1.18 ± 1.44	2.36 ± 5.90	0.039

*Compared *via* paired *t*-test; *p* < 0.05 is considered statistically significant. ^18^F-FDG, ^18^F-fluorodeoxyglucose positron emission tomography; L, left; R, right.

## 4. Discussion

Compared with the findings of previous studies, the present work revealed concrete sub-regional and lateralized changes in metabolic characteristics in patients with anti-LGI1 encephalitis based on ^18^F-FDG PET images. Asymmetric hypo- or hypermetabolism as well as differences in positional uptake in certain brain structures were observed, indicating that some brain regions are more likely to be affected by autoimmune encephalitis. These results could provide clues for interpreting various clinical symptoms.

Previous studies have shown that abnormalities were more likely to be found on brain ^18^F-FDG PET images compared to MRI in patients with autoimmune encephalitis (16–18). As for anti-LGI1 encephalitis, increased metabolism in the hippocampus, basal ganglia, and medial temporal lobe has been reported to be associated with hypometabolism within cortical areas, such as the parietal, frontal, and occipital lobes, the cingulate cortex, and the paracentral lobule (12, 15, 17, 19). The related clinical symptoms include memory impairment, seizures, pyramidal signs, emotional disorders, behavioral changes, cognitive deficits, and impaired language function. Furthermore, in our previous study, we showed that increased metabolism in the basal ganglia and the hippocampus associated with decreased cortical metabolism was a general metabolic alteration observed in patients with anti-LGI1 encephalitis (15). In addition, the extent of the metabolic abnormalities between the cortical and subcortical areas was associated with the degree of neurological disorder. In this study, almost all of the cerebral areas of interest demonstrated a decreased metabolic pattern compared to that of the pons; however, the medial temporal region was prone to hypermetabolism, a finding that is consistent with those of several previous studies (13, 14, 17). In addition to the general cerebral lobe regions, some brain substructures, such as the cingulate gyrus, sensorimotor cortex, precuneus, and primary visual cortex, were shown to be affected by hypometabolism in the present anti-LGI1 encephalitis sample. These substructures play an important role in mediating physiological functions, including episodic memory, cognition, emotional control, stimulation of motor movement, information processing, and hallucinations (20–23). A decrease in ^18^F-FDG metabolism in these substructures can trigger physiological disorders. Identifying the metabolic abnormalities in these brain regions might provide new clues for understanding and interpreting the clinical presentation of patients with anti-LGI1 encephalitis.

The presence of concurrent hypermetabolism in the hippocampus and striatum with neocortical hypometabolism was shown to be a conventional metabolic abnormality in those with anti-LGI1 encephalitis in our previous findings (15). Similarly, the present study demonstrated increased metabolism in the medial temporal region (13/13, 100%) and bilateral basal ganglia (11/13, 84.6%) in association with decreased metabolism in at least one of the brain areas of interest (13/13, 100%). Hypermetabolism in the striatum has been reported to be a sign for early-stage encephalitis, which is evident before other clinical diagnostic means (8, 13). This change in activity was positively observed in the enrolled patients, with or without bilateral thalamic hypermetabolism. In addition, hypermetabolism in the basal ganglia has been shown to be most prominent in patients with FBDS; which is characteristic of anti-LGI1 encephalitis (17, 19). All patients who experienced FBDS in this study presented with hypermetabolism in the basal ganglia. FBDS is regarded as a typical presentation at the early stage of anti-LGI1 encephalitis, presented as dystonia on the same side of the face, upper or lower limb, and accompanied by increased metabolism of ^18^F-FDG in the basal ganglia. On one hand, for such movement disorders as Parkinson’s disease, increased glucose metabolism in globus pallidus, thalamus, pons and cerebellum was observed, as well as decreased glucose metabolism in pre-motor area and posterior parietal area; on the other hand, abnormal or typical electroencephalogram pattern in patients with seizures is usually absent when FBDS occurs, and its reaction to antiepileptic drugs remains unsatisfactory. In consideration of the above evidence, FBDS is tended to be regard as a type of movement disorder that may be attributed to the deep structure impairment of the brain. However, this issue remains controversial which needs further investigation and convincing evidence.

An asymmetric trend in metabolic activity between the bilateral cerebral regions was observed in this study, although the hyper- or hypometabolic asymmetry within each brain region varied. For the prefrontal lobe, temporal lobe, and sensorimotor cortex, the right side was more likely to exhibit greater uptake compared with the opposite side, regardless of whether there was hyper- or hypometabolism. In contrast, the occipital lobe and precuneus presented an opposite asymmetric pattern; however, these patterns were not homogeneous in other brain regions such as the cingulate cortex and the parietal lobes. Among all of the brain areas of interest, the metabolic activity of the right posterior cingulate was significantly lower than that of the opposite side, whereas the rest of the areas failed to demonstrate significant differences. It has been reported that in patients with anti-LGI1 encephalitis, ^18^F-FDG PET uptake is asymmetrical in the basal ganglia and hippocampus (9). In the present study, ^18^F-FDG metabolic asymmetry was evident in several brain substructures, indicating that this interesting phenomenon is not restricted to the brain areas where metabolic asymmetry has been previously described.

The differences in LGI1 expression were specifically localized to deep structures, and it would have been difficult to characterize changes in the cortical regions. As ^18^F-FDG is not a direct indicator of LGI1 protein expression, the present findings of metabolic asymmetry in cortical regions could have been explained as a secondary effect driven by the asymmetric alterations within deep structures. LGI1 antibody expression in the hippocampus and neocortex has been reported in another study (24), which could partially explain the asymmetric metabolic patterns observed in the cortical regions. There is existing evidence to suggest that LGI1 takes part in brain development in rodents (25, 26) and that the LGI1 protein could act as one of the key factors determining metabolic asymmetry (11). Asymmetry of cell architecture and of cell migration during development could probably be a result of differences in LGI1 expression levels (27). However, the underlying mechanisms driving metabolic asymmetry remain to be elucidated, making interpretation of these findings difficult.

In addition to the asymmetric metabolic pattern of cortical regions in anti-LGI1 encephalitis, we further compared the metabolic activity within single brain regions between hemispheres. In the prefrontal and temporal lobes, the medial cortical region demonstrated significant hypermetabolism compared to that of its lateral counterpart in both the left and right hemispheres. Similarly, metabolic activity in the anterior cingulate cortex was significantly higher than that in the posterior cingulate cortex on both sides. A previous study found evidence of T-cell infiltration in the amygdala and hippocampus (28), and T cell-mediated cytotoxicity can lead to increased ^18^F-FDG uptake in the corresponding brain area (29). Furthermore, increased glucose uptake by neurons and glial cells due to the binding of LGI1 antibodies could be detected by ^18^F-FDG PET, which could indirectly reflect alterations in LGI1 protein expression and functional connectivity between the deep structures and cortical regions. Accordingly, hypermetabolism in specific regions in anti-LGI1 encephalitis is likely to indicate the presence of antibody-induced neuronal disturbance and the subsequent impairment of neurological function.

The medial prefrontal cortex integrates and acts as a convergence point for updated information through its connections with other cortical and subcortical areas (30). It includes the medial precentral area, anterior cingulate cortex, prelimbic cortex, and infralimbic cortex, with the latter two receiving functional input from the ventral hippocampus, basolateral amygdala, midline thalamus, and contralateral medial prefrontal cortex. A better understanding of these physiological circuits would provide clues for interpreting the significantly higher metabolism in the medial prefrontal cortex and anterior cingulate cortex compared with that of their lateral/posterior counterparts. Moreover, these regional differences in ^18^F-FDG metabolism within a cerebral hemisphere could be observed on the opposite side as well.

However, for the parietal lobe, a significant difference in metabolic activity between the superior and inferior cortical areas was only observed in the left hemisphere. The temporoparietal connections between the superior temporal gyrus and the inferior parietal lobule, as part of the superior longitudinal fasciculus/arcuate fasciculus, have been investigated in previous studies. One such study demonstrated that the connections between the posterior temporal lobe and the superior parietal lobule mediate key processes such as memory, attention, emotional modulation, and language (31). It has been suggested that there are close functional connections between the parietal and temporal lobes. The superior parietal cortex is essential for the manipulation and rebuilding of memory information (32), and the inferior parietal lobule is predominantly known for its role in visuospatial processing. In this study, the symptoms presented in the enrolled patients were more closely related with functional impairment of the superior parietal cortex. As a result, on one hand, the superior parietal cortex, which exhibited LGI1-induced functional impairment, was more likely to exhibit a relatively higher degree of ^18^F-FDG metabolism compared to that of the inferior parietal lobule. On the other hand, the relatively higher metabolism in the superior parietal cortex might have been a secondary manifestation resulting from the alterations in the deep structure located in the temporal lobe. This significant metabolic difference was not observed on the right side. The metabolic alteration in the substructure of the parietal lobe requires further verification through a robust study with a larger sample size.

There were several limitations in the present study. First, this was a retrospective study that took place in a single institution, which could have made it liable to selection bias. Second, a relatively small sample size might have limited the ability to detect certain metabolic differences between brain regions or sides. As there was no fixed difference value to determine the abnormal glucose metabolism alterations, the effect size for conducting power analysis was not available before initiating the study. We have calculated the effect size and proper sample size for each comparison group based on the data in this study to serve as reference for further studies (Supplementary Table 2). Meanwhile, the findings in this study require further verification. Third, the semiquantitative approach to image analysis using Cortex ID software did not generate metabolic data corresponding to the deep nuclei. Finally, the handedness factor was not assessed in the present study to perform the metabolism analysis between dominant and non-dominant hemispheres. Further prospective studies are required to fully characterize the differences in metabolic patterns in anti-LGI1 encephalitis.

Overall, the present study suggests an asymmetric ^18^F-FDG metabolic pattern in patients with anti-LGI1 encephalitis. Moreover, significant regional metabolic differences were revealed in the prefrontal and temporal lobes bilaterally, as well as in the cingulate cortex. Further prospective, multicenter studies with larger patient populations are warranted to confirm these findings.

## Data availability statement

The original contributions presented in the study are included in the article/Supplementary marerial, further inquiries can be directed to the corresponding author.

## Ethics statement

The studies involving human participants were reviewed and approved by the Ethics Committee of Beijing Tiantan Hospital, Capital Medical University. Written informed consent for participation was not required for this study in accordance with the national legislation and the institutional requirements.

## Author contributions

KW: analysis and interpretation of data, and drafting manuscript. XZ and LY: recruitment, diagnosis, and patient assessment. QC and QW: interpretation of data and revision of the manuscript. LA: design and conceptualization of the study, and revision of the manuscript. All authors contributed to the article and approved the submitted version.

## Funding

This work was supported by funds from the National Natural Science Foundation of China (82001769).

## Conflict of interest

The authors declare that the research was conducted in the absence of any commercial or financial relationships that could be construed as a potential conflict of interest.

The handling editor JL declared a shared affiliation, though no other collaboration, with authors KW, XZ, LY, QC, QW, and LA at the time of the review.

## Publisher’s note

All claims expressed in this article are solely those of the authors and do not necessarily represent those of their affiliated organizations, or those of the publisher, the editors and the reviewers. Any product that may be evaluated in this article, or claim that may be made by its manufacturer, is not guaranteed or endorsed by the publisher.
