# Supplementary material for: Cortical metabolic characteristics of anti-leucine-rich glioma-inactivated 1 antibody encephalitis based on 18F-FDG PET
Source: Front Neurol. 2023 Mar 31;14:1100760. doi: 10.3389/fneur.2023.1100760 (PMC10102654; doi:10.3389/fneur.2023.1100760)
Supplement: Supplementary file 1 [file Table_1.DOCX]

**Supplemental Table 1 ^18^F-FDG PET findings among patients with anti-LGI1 encephalitis**

| Patient  no. | ^18^F-FDG PET findings | | | | | Brain CT/MRI interpretation |
| --- | --- | --- | --- | --- | --- | --- |
|  | Visual PET interpretation | Semiquantitative analysis (3D-SSP) * | | | Basal ganglia and/or thalamus metabolism |  |
|  |  | Cortical regions with significant metabolic changes | | Interpretation |  |  |
|  |  | Hypometabolism | Hypermetabolism |  |  |  |
| 1 | Abnormal | PS (R) | – |  | Hypermetabolism | Normal (CT) |
| 2 | Abnormal | – | **PFM (R)**, **AC (R), TM (R, L)** | Abnormal | Hypermetabolism | Normal (CT) |
| 3 | Abnormal | PFL (R, L), PFM (R), PC (R), P (R), **PI** (R, **L)** | – | Abnormal | Hypermetabolism | Normal (CT) |
| 4 | Abnormal | **PFL (R, L), PFM (R, L), SM (R, L)**, AC (R, L), **PC (R, L), P (R, L), PS (R, L), PI (R, L), OL (R, L), TL (R, L)** | – | Abnormal | Hypermetabolism | Normal (CT) |
| 5 | Abnormal | **PFL (R, L), PFM (R, L)**, PC (R), **P (R, L)**, **PS** (R, **L)**, **PI (R, L)**, OL (R, L), PV (R), **TL (R, L),** TM (R) | – | Abnormal | Hypermetabolism | Normal (CT) |
| 6 | Abnormal | – | – | Normal | Hypermetabolism | Normal (CT) |
| 7 | Abnormal | **PFL (R, L)**, PFM (R, L), PC (R, L), **P (R, L)**, PS (R), **PI (R**, L), OL (R, L), **PV (R, L)**, TL (R) | **TM (R, L)** | Abnormal | Hypermetabolism | Normal (CT) |
| 8 | Abnormal | **PFL (R, L)**, **PFM (R, L)**, SM (R, L), **AC (R)**, **PC (R, L)**, **P (R**, L), **PS (R, L), PI (R, L)**, **OL (R**, L), **PV (R**, L), **TL (R, L)** | **TM (L)** | Abnormal | Hypermetabolism | Normal (CT) |
| 9 | Abnormal | PS (L), PI (L), | PV (L) | Abnormal | Normal | Abnormal (MRI) |
| 10 | Abnormal |  | **TM (R)** | Abnormal | Normal | Normal (CT) |
| 11 | Abnormal | **PFL (R, L)**, PFM (R, L), **PC (R, L)**, **P (L)**, **PI (R, L)**, **OL (L)**, | **TM (R, L)** | Abnormal | Hypermetabolism | Normal (CT) |
| 12 | Abnormal | – | – | Normal | Hypermetabolism | Normal (CT) |
| 13 | Abnormal | PFL (L), **P (R**, L), **PS (R, L)**, PI (R, L), **OL (R**, L), PV (R, L), | – | Abnormal | Hypermetabolism | Normal (CT) |
|  | | | | | | |

*An average z-score > 2 (two-tailed P=0.05) was considered to be indicative of a significant metabolic change. Regions in bold font represent areas with higher absolute z-scores (absolute z > 2.58, two-tailed P=0.01).

^18^F-FDG, ^18^F-fluorodeoxyglucose positron emission tomography; LGI1, leucine-rich glioma-inactivated 1; CT, computed tomography; MRI, magnetic resonance imaging; 3D-SSP = 3D stereotactic surface projections; R, right; L, left; PFL = prefrontal lateral; PFM = prefrontal medial; SM = sensorimotor; AC = anterior cingulate; PC = posterior cingulate; P = precuneus; PS = parietal superior; PI = parietal inferior; OL = occipital lateral; PV = primary visual; TL = temporal lateral; TM = temporal medial.
