# Supplementary material for: Cortical metabolic characteristics of anti-leucine-rich glioma-inactivated 1 antibody encephalitis based on 18F-FDG PET
Source: Front Neurol. 2023 Mar 31;14:1100760. doi: 10.3389/fneur.2023.1100760 (PMC10102654; doi:10.3389/fneur.2023.1100760)
Supplement: Supplementary file 2 [file Table_2.DOCX]

**Supplemental Table 2 Estimated sample size and effect size for paired t-test in this study**

| Comparison groups | Effect size (d) | Estimated sample size* |
| --- | --- | --- |
| Prefrontal **lateral** R-L | 0.18 | 245 |
| Prefrontal **medial** R-L | 0.10 | 787 |
| Prefrontal **R** lateral-medial | 1.03 | 10 |
| Prefrontal **L** lateral-medial | 1.09 | 9 |
| **Sensorimotor** R-L | 0.20 | 199 |
| **Anterior** cingulate R-L | 0.11 | 651 |
| **Posterior** cingulate R-L | 0.63 | 22 |
| Cingulate **R** anterior-posterior | 1.22 | 8 |
| Cingulate **L** anterior-posterior | 0.61 | 24 |
| **Precuneus** R-L | 0.30 | 90 |
| Parietal **superior** R-L | 0.42 | 47 |
| Parietal **inferior** R-L | 0.28 | 103 |
| Parietal **R** superior-inferior | 0.08 | 1229 |
| Parietal **L** superior-inferior | 0.64 | 22 |
| **Occipital lateral** R-L | 0.05 | 3142 |
| **Primary visual** R-L | 0.35 | 67 |
| Temporal **lateral** R-L | 0.56 | 28 |
| Temporal **medial** R-L | 0.19 | 220 |
| Temporal **R** lateral-medial | 0.64 | 22 |
| Temporal **L** lateral-medial | 0.96 | 11 |

*Number of pairs, significant level=0.05, power(1-β)=0.8, 2-tail.
